# Draft Genome Sequence of *Kluyvera* *intestini* Strain GT-16 Isolated from the Stomach of a Patient with Gastric Cancer

**DOI:** 10.1128/genomeA.01432-16

**Published:** 2016-12-22

**Authors:** George Tetz, Victor Tetz

**Affiliations:** Human Microbiology Institute, New York, New York, USA

## Abstract

Here, we report the complete genome sequence of the novel, non-spore-forming *Kluyvera intestini* strain GT-16, isolated from the stomach of a patient with gastric cancer. The genome is 5,868,299 bp in length with a G+C content of 53.0%. It possesses 5,350 predicted protein-coding genes encoding virulence factors and antibiotic resistance proteins.

## GENOME ANNOUNCEMENT

The genus *Kluyvera* comprises aerobic or facultatively anaerobic, Gram-negative rods, motile with peritrichous flagella. *Kluyvera* spp. have been found in water, soil, and hospital environments (1). They have also been isolated from human clinical specimens such as urine, stool, and blood and are considered clinically significant pathogens (2–4).

In this study, *Kluyvera intestini* strain GT-16 was isolated from the stomach of a patient with gastric cancer. The complete sequence of its 16S rRNA gene displayed 99% similarity with that of *K. intermedia*, *Yokenella regensburgei*, and *Enterobacter lignolyticus*.

The isolate was identified as *K. intermedia*, with very good species identification, by using Vitek version 2 according to the manufacturer’s instructions (5). Whole-genome sequencing was performed using the Illumina HiSeq 2500 platform (Illumina GA IIx, Illumina, USA). A draft genome was assembled using SPAdes version 3.5.0 with a 110-fold average coverage (6).

Eventually, 137 scaffolds were obtained, consisting of 5,868,299 bp with an overall G+C content of 53.0%. Gene annotation using NCBI the Prokaryotic Genome Annotation Pipeline resulted in the identification of 5,350 protein-coding sequences, 90 tRNA genes, seven rRNAs, and 16 noncoding RNA operons (7).

Several virulence-related genes, such as those coding for hemolysin D, peptidases, flagellar proteins, and exonucleases, were present in *K. intestini* strain GT-16 (8). The genome carries genes of multidrug resistance efflux pumps and genes encoding resistance to antibiotics, including macrolides, quaternary ammonium compounds, tellurium, and bleomycin, and beta-lactamase genes.

According to DNA-DNA hybridization prediction based on genome BLAST distance phylogeny, the genetic distance between the *K. intestini* strain GT-16 genome and the *K. intermedia* ASM 102213v1,* K. intermedia* NBRC 102594*, Y. regensburgei* ATCC 49455, and *E. lignolyticus* SCF1 genomes were estimated, and DNA-DNA hybridization values of 45.50%, 21.70%, 21.70%, and 22.10%, respectively, were calculated in the genome-to-genome distance calculator web server version 2.0. These similarity values are known to be below the threshold of 70% for genomes belonging to the same species (9, 10).

The genome sequence analysis of *K. intestini* strain GT-16 could help contribute to determining the role of *Kluyvera* spp. as human pathogens and could provide insights into the microbiota composition of gastric cancer patients.

### Accession number(s).

This complete genome sequencing project has been deposited in GenBank under the accession number MKZW00000000.
